# Leveraging the Social Determinants of Health: What Works?

**DOI:** 10.1371/journal.pone.0160217

**Published:** 2016-08-17

**Authors:** Lauren A. Taylor, Annabel Xulin Tan, Caitlin E. Coyle, Chima Ndumele, Erika Rogan, Maureen Canavan, Leslie A. Curry, Elizabeth H. Bradley

**Affiliations:** 1 Department of Health Policy and Management, Harvard Business School, Boston, Massachusetts, United States of America; 2 Department of Health Policy and Management, Yale School of Public Health, New Haven, Connecticut, United States of America; The Chinese University of Hong Kong, HONG KONG

## Abstract

We summarized the recently published, peer-reviewed literature that examined the impact of investments in social services or investments in integrated models of health care and social services on health outcomes and health care spending. Of 39 articles that met criteria for inclusion in the review, 32 (82%) reported some significant positive effects on either health outcomes (N = 20), health care costs (N = 5), or both (N = 7). Of the remaining 7 (18%) studies, 3 had non-significant results, 2 had mixed results, and 2 had negative results in which the interventions were associated with poorer health outcomes. Our analysis of the literature indicates that several interventions in the areas of housing, income support, nutrition support, and care coordination and community outreach have had positive impact in terms of health improvements or health care spending reductions. These interventions may be of interest to health care policymakers and practitioners seeking to leverage social services to improve health or reduce costs. Further testing of models that achieve better outcomes at less cost is needed.

## Introduction

Social determinants of health have taken center stage in recent health policy discussions, particularly with the growing emphasis on global payment, accountable care organizations (ACO)[1], and other initiatives focused on improving population health. Health care providers are increasingly being asked to measure impact in terms of the health outcomes of the population they serve. Given that medical care influences a relatively small portion of overall health [2, 3], ACO and value-based financing models face substantial challenges in equipping health care providers to achieve improvements in the population’s health.

Many researchers have examined the relative contributions of health care services, genetics, behaviors, environment and social factors in promoting health and reducing premature mortality [3–6]. Overwhelmingly, studies find that non-medical factors including social, behavioral and environmental determinants of health consistently play a substantially larger role than medical factors. Similar patterns hold for specific health outcomes, including burdensome, high-cost diseases such as heart disease, stroke and diabetes [7–9], although the relative contributions may vary by 5–10 percent depending on the health outcome in question.

Despite the evidence, an enduring challenge of the social determinants of health literature has been translating its insights into actionable recommendations. The literature is replete with studies dating to the 1970s that indicate that poor social determinants of health are harmful to health both in the short and longer-term [10–14] as well as a growing body of literature which demonstrates positive impact of favorable social conditions on health outcomes [15–17]. Nevertheless, the literature has not yet been reviewed comprehensively to generate an integrated, evidence-based summary of how to best address the social determinants to achieve positive health effects without increasing, and perhaps even decreasing, health care spending. Accordingly, we sought to synthesize the existing empirical evidence about the impact of social service interventions on health outcomes and health care spending, with particular attention to identifying programs and practices that achieved both improvements in health as well as potential reductions in health care spending.

## Methods

We summarized the peer-reviewed literature that examined the impact of investments in social services or investments in integrated models of health care and social services on health outcomes and health care spending. We used the PubMed database to execute our initial search and included relevant literature published in English between January 2004 and October 2014. We ran a number of search strings comprised of a combination of social and health keywords. Social service keywords included were: “social service,” “social spending,” “social welfare,” “housing,” “education,” “income support,” “nutrition,” “food stamp,” “SNAP,” “public safety,” and “transportation.” Health and health care keywords were: “health,” “health outcomes,” “health saving,” “health costs,” and “health spending,” and “health expenditure.” Eligibility criteria included: 1) inclusion of a social service intervention, or a health care intervention that specifically targeted a social, behavioral, or environmental determinant of health, 2) quantitative measurement of a health outcome, health care costs, or both; and 3) well-documented study design. We also reported utilization outcomes such as hospital admissions and emergency department visits, as these can influence health care spending. We excluded papers that examined health behaviors (e.g., cigarettes smoked, steps walked) rather than health outcomes.

**Fig 1:**
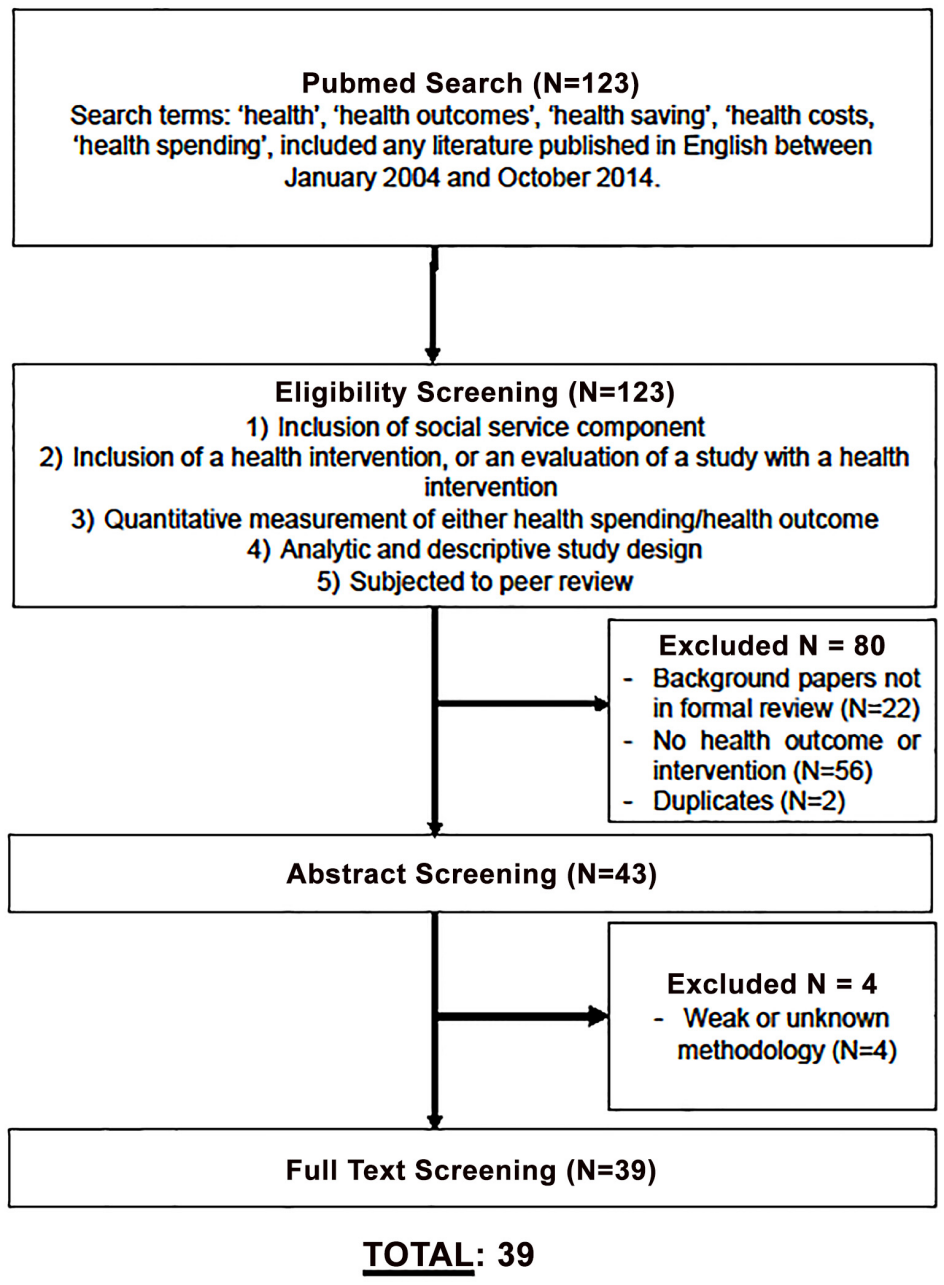
Sampling Schematic.

The search yielded 123 unique articles. Screening and analysis was conducted by three members of the research team (LT, CC, CN) who met frequently to review decisions and disagreements, which were resolved through negotiated consensus. A total of 80 of the 123 studies were excluded for not meeting eligibility criteria based on a review of their abstracts, leaving 43 articles for full article review. Upon further investigation, the review of the 43 full-length articles resulted in exclusion of an additional 4 articles, yielding a sample of 39 articles for analysis (Fig 1). These 39 articles were reviewed independently by the three members of the research team (LT, CC, CN) to record data on study design, sample characteristics, geographic location, description of the social service intervention, and empirical findings related to intervention-associated changes in health outcomes or health care spending. Because the 39 articles reported a range of health outcomes and cost metrics, the data were unable to accommodate a statistical meta-analysis.

## Results

### Overview

Of the 39 articles, 32 (82%) reported some significant positive effects on either health outcomes (N = 20), health care costs (N = 5), or both (N = 7). Of the remaining 7 (18%) studies, 3 had non-significant results, 2 had mixed results, and 2 had negative results in which the interventions were associated with poorer health outcomes. No particular study design, intervention, or population was recurrent among studies with non-significant, mixed, and negative findings. For instance, one of the studies with negative findings was a randomized controlled trial evaluating a housing intervention on the mental health of male adolescents [18] while the other was cross-sectional study evaluating SNAP participation on the Body Mass Index of low-income adults [19].

Among the 32 studies with positive outcomes, 10 (31%) were related to housing support, 7 (22%) to nutritional support, 4 (13%) to income support, 8 (25%) to care coordination and community outreach programs, and 3 (9%) to other types of interventions (Table 1). Study designs included cohort studies (N = 15), randomized controlled trials (N = 12), cross-sectional (N = 4), pre-post interventions (N = 6), quasi-experimental (N = 1), and post-test only evaluation (N = 1). We identified no studies explicitly examining the health effects of transportation or public safety interventions. Approximately 72% of all studies included in this review focused on low-income populations. We have summarized key findings and associations between programs and health outcomes/spending in greater depth (Table 2).

**Table 1:** Summary of findings in the literature (N = 39).

Findings	Housing Support N (%)	Nutrition Support N (%)	Income Support N (%)	Care coordination and community outreach N (%)	Other* N (%)	Total N (%)
**Positive, significant findings**						
**Positive health outcomes**	5 (42%)	7 (64%)	3 (75%)	2 (22%)	3 (100%)	20 (51%)
**Reduced costs**	1 (8%)	0 (0%)	0 (0%)	4 (44%)	0 (0%)	5 (13%)
**Both health outcomes and reduced costs**	4 (33%)	0 (0%)	1 (25%)	2 (22%)	0 (0%)	7 (18%)
**Other findings**						
**Mixed results**	0 (0%)	1 (9%)	0 (0%)	1 (9%)	0 (0%)	2 (5%)
**Non-significant effects**	1 (8%)	2 (18%)	0 (0%)	0 (0%)	0 (0%)	3 (8%)
**Negative health outcomes**	1 (8%)	1 (9%)	0 (0%)	0 (0%)	0 (0%)	2 (5%)
**Total**	12 (100%)	11 (100%)	4 (100%)	9 (100%)	3 (100%)	39 (100%)

*Other studies contained interventions that had major educational components that were associated with improved health outcomes, especially among children.

**Table 2:** Summary of associations between programs and health outcomes/spending.

INTERVENTION TYPE	AUTHOR, TITLE	YEAR	ASSOCIATION BETWEEN INTERVENTION AND HEALTH OUTCOMES/SPENDING^1^ (+/-)	STUDY LOCATION	STUDY DESIGN, SAMPLE TYPE AND SIZE	KEY FINDINGS RELATED TO HEALTH OUTCOMES OR COST SAVINGS
**Housing Support**	**Barton, A., Basham, M., Foy, C., Buckingham, K., & Somerville, M,** *The Watcombe Housing Study*: *the short term effect of improving housing conditions on the health of residents*	2007	Significant	United Kingdom	**Intervention:** Housing improvements, including central heating, ventilation, rewiring, and re-roofing. Participants were also given a booklet explaining uses of new equipment. **Study design:** Randomized to waitlist controlled trial, 12 months foll0w-up.**Study sample:** 480 public housing residents	**Reported Outcomes:** Health outcomes. For those living in intervention houses, non-asthma related chest problems (Man-Whitney, p = 0.005) and combined asthma symptom score (Mass-Whitney, z = 2.7, p = .007) diminished significantly compared to control houses.
	**Basu, A., Kee, R., Buchanan, D., & Sadowski, L. S.,** *Comparative Cost Analysis of Housing and Case Management Program for Chronically Ill Homeless Adults Compared to Usual Care*	2011	Non-significant	Chicago, IL, USA	**Intervention**: Housing and case management program**Study design**: Randomized controlled trial**Study sample**: 407 homeless adults with chronic medical illnesses	**Reported Outcomes**: Cost savings. The intervention group generated a cost savings of $6307 per person (p = 0.23). Among those who were chronically homeless, there was an associated cost saving of $9809 and among those living with HIV, $6622.
	**Castle, N., & Resnick, N.,** *Service-Enriched Housing The Staying at Home Program*.	2014	Significant	Pittsburgh, PA, USA	**Intervention:** Service-enriched housing comprised of care coordination, advance planning, medication management and a healthcare diary. **Study design:** Pre-post intervention. **Study sample:** 1135 low-income older adults in publicly subsidized buildings	**Reported Outcomes**: Health outcomes and cost savings.Among the intervention group, 7/10 desired outcomes were achieved: increase in healthcare utilization (p <.001); improved self-reported health (p <.05), number of health conditions (p <.05), more non-institutional services (p <.01), lower likelihood of institutionalization (p <.001), less ER visits (p <.001) and less unplanned hospitalizations (p <.01). For every 100 residents in the program approximately four nursing home transfers were avoided.
	**Edwards, R. T., Neal, R. D., Linck, P., Bruce, N., Mullock, L., Nelhans, N., … & Woodfine, L**, *Enhancing ventilation in homes of children with asthma*: *cost effectiveness study alongside randomized control trial*	2011	Significant	United Kingdom	**Intervention:** Housing modifications designed to improve ventilation and household heating. **Study design:** Randomized controlled trial, 12 months follow-up **Study sample**: Children (5–14 years) with moderate or severe asthma (n = 177)	**Reported Outcomes:** Health outcomes and cost savings. The intervention reduced the level of asthma severity, shifting 17% of children in the intervention group from ‘severe’ to ‘moderate’ asthma, compared with a 3% shift in the control group. On average, the intervention cost £1718 per child treated. After bootstrapping, results yielded an incremental cost effectiveness ratio (ICER) of £234 per point improvement (95% CI: £140- £590) on the 100 point Peds-QL asthma specific scale. For asthmatic children, the ICER was £165 (£84-£424) for children with severe asthma.
	**Garland, E., Steenburgh, E. T., Sanchez, S. H., Geevarughese, A., Bluestone, L., Rothenberg, L., … & Foley, M.,** *Impact of LEED-certified affordable housing on asthma in the South Bronx*.	2013	Significant	New York, New York, USA	**Intervention:** Housing complex, home-based educational module. **Study design:** Pre/post intervention, 18 months follow-up **Study sample:** Affordable housing residents in the South Bronx, New York (n = 18)	**Reported Outcomes:** Health outcomes and cost savings. Between the intervention and control group, there was a decrease in continuous daily respiratory symptoms (p<0.001), asthma symptoms disrupting sleep in the past month (p = 0.028), and urgent visits to a healthcare professional in the past 3 months (p = 0.038).
	**Hawk, M., & Davis, D.,** *The effects of a harm reduction housing program on the viral loads of homeless individuals living with HIV/Aids*.	2012	Significant	USA	**Intervention:** Harm reduction housing first model. **Study design**: Pre-post intervention, follow up of 15 months. **Study sample**: Homeless individuals living with HIV/AIDS (n = 26)	**Reported Outcomes:** Health outcomes. There was a significant difference between undetectable viral load measures at baseline and at follow-up (27% vs 69%, p = 0.02). The median time it took for residents to reach undetectable viral load was 116 days or 3.9 months.
	**Jacobs DE, Breysse J, Dixon SL, Aceti S, Kawecki C, James M, Wilson J.,** *Health and housing outcomes from green renovation of low-income housing in Washington*, *DC*	2014	Significant	Washington, DC, USA	**Intervention:** Housing renovation in accordance with the Green Health Housing Improvements that include integrated design, location and neighborhood fabric, site, water conservation, use of sustainable materials, and healthy living environments. **Study design:** Pre/post study design with no control group, 1 year follow-up from baseline**. Study sample:** Low-income housing residents in Washington DC (n = 57 adults; n = 64 children)	**Reported Outcomes**: Health outcomes. Self-reported general health in adults significantly improved from 59% to67% (p = .026). There were also large statistically significant improvements in water/ dampness problems, cockroaches and rodents, and reduced pesticide use.
	**Kessler, R. C., Duncan, G. J., Gennetian, L. A., Katz, L. F., Kling, J. R., Sampson, N. A., … & Ludwig, J**., *Associations of Housing Mobility Interventions for Children in High-Poverty Neighborhoods With Subsequent Mental Disorders During Adolescence*.	2014	Non-significant	Boston, MA	**Intervention**: Housing mobility interventions: low-poverty voucher group, and a traditional voucher group. The low-poverty voucher group received vouchers to move to low-poverty neighborhoods with enhanced mobility counseling, whereas the traditional voucher group received geographically unrestricted vouchers. **Study design**: Randomized controlled trial from 1994–1998, with follow-up in June 2008 –April 2010.**Study sample**: 4604 public housing families with 3689 children in high-poverty neighborhoods. Children were aged 13-19years at end of follow-up (0–8 years at randomization).	**Reported Outcomes**: Health outcomes. The study investigated mental health outcomes, including major depressive disorder, panic disorder, posttraumatic stress disorder (PTSD), oppositional-defiant disorder, intermittent explosive disorder, and conduct disorder. Boys in the low-poverty voucher group had significantly greater rates of depression compared to the control group (OR: 2.2, 95% CI: 1.2–3.9), as well as greater rates of conduct disorder (OR: 3.1, 95%: 1.7–5.8). However, girls in the traditional voucher group had decreased rates of major depression (OR: 0.6, 95%: 0.3–0.9) and conduct disorder (OR: 0.1, 95% CI: 0.0–0.4).
	**Larimer, M. E., Malone, D. K., Garner, M. D., Atkins, D. C., Burlingham, B., Lonczak, H. S., … & Marlatt, G. A.,** *Health Care and Public Service Use and Costs Before and After Provision of Housing for Chronically Homeless Persons With Severe Alcohol Problems*	2009	Significant	Seattle, WA	**Intervention:** the “Housing First” intervention. **Study design**: Quasi-experimental design comparing housed and wait-listed participants enrolled between Nov 2005 and Mar 2007. **Study sample**: 95 housed participants (drinking permitted) and 39 wait-list control participants.	**Reported Outcomes:** Cost savings. Housing First participants incurred a median cost of $4066 per person per month in the first year prior to the study. Median monthly costs decreased to $1492 and $958 after 6 and 12 months in housing. There was a total cost rate reduction of 53% for housed participants relative to wait-list controls.
	**Ludwig, J., Sanbonmatsu, L., Gennetian, L., Adam, E., Duncan, G. J., Katz, L. F., … & McDade, T. W.,** *Neighborhoods*, *obesity*, *and diabetes—a randomized social experiment*.	2011	Mixed	Baltimore, MD; Boston, MA; Chicago, IL; Los Angeles CA; and New York City, NY.	**Intervention:** Housing vouchers that were redeemable only if participants moved to a low-poverty census tract, and unrestricted traditional vouchers. **Study design:** Randomized controlled trial. **Study sample:** 4498 women with children living in public housing in high poverty urban census tracts, from 1994–1998.	**Reported Outcomes:** Health outcomes The prevalences of BMI of 53 or more, a BMI, of 40 or more, and a glycated hemoglobin level of 6.5% or more were significantly *lower* in the group receiving the low-poverty vouchers than in the control group, with an absolute difference of 4.61 percentage points, 3.38, and 4.31 respectively.
	**Rantz, M., Popejoy, L. L., Galambos, C., Phillips, L. J., Lane, K. R., Marek, K. D., … & Ge, B.** *The continued success of registered nurse care coordination in a state evaluation of aging in place in senior housing*.	2014	Significant	Missouri, USA	**Intervention:** Aging in Place (AIP) Delivery Care model. **Study design**: Post-intervention evaluation (4 years) **Study sample**: 128 older adults in Missouri (long-term care insurance and private pay).	**Reported Outcomes**: Health outcomes and cost savings. The AIP intervention has positive health outcomes for participants: fall risk (27 v. 20.9, gait velocity (66.5 v. 74.5), Functional Ambulation Profile (72.9 v. 74), handgrips (rt. 19 v. 24), Short-Form 12 Physical Health (38 v 39), Short-Form 12 Mental Health (54.7 v. 38.7).Combined care and housing costs for any resident who was receiving additional care services and qualified for nursing home care (n = 44) was about $20,000 less per year per person than nursing home care.
	**Szanton, S. L., Thorpe, R. J., Boyd, C., Tanner, E. K., Leff, B., Agree, E., … & Gitlin, L. N.,** *Community Aging in Place*, *Advancing Better Living for Elders*: *A Bio‐Behavioral‐Environmental Intervention to Improve Function and Health‐Related Quality of Life in Disabled Older Adults*.	2011	Significant	USA	**Intervention**: A multi-component behavior and home repair intervention; including occupational therapy, nursing, handyman repairs (CAPABLE)**Study design:** Prospective Randomized controlled trial, 6 months follow-up. **Study sample**: 40 low-income older adults with difficulties in one or more activities of daily living (ADL), or 2 or more instrumental activities of daily living (IADLs).	**Reported Outcomes:** Health outcomes The intervention group improved upon all recorded outcomes. Comparing mean change in the intervention group compared to the control group, the CAPABLE intervention had an effect size of 0.63 for reducing difficulty in ADLs, 0.62 for reducing difficulty in IADLS, 0.89 for quality of life, and 0.55 for falls-efficacy.
**Nutritional Support**	**El-Bastawissi, A. Y., Peters, R., Sasseen, K., Bell, T., & Manolopoulos, R.** *Effect of the Washington special supplemental nutrition program for women*, *infants and children (WIC) on pregnancy outcomes*.	2007	Significant	Washington state, USA	**Intervention**: Washington State Special Supplemental Nutrition Program for Women, Infants and Children (WIC).**Study design:** Retrospective-linkage cohort study **Study sample**: 42495 Women who enrolled in Washington WIC from 9/1/1999 to 12/31/2000.	**Reported Outcomes**: Health outcomesWIC was protective for preterm delivery, being particularly protective for women with abortion and inadequate prenatal care (OR = 0.4; 95% CI: 0.3–0.5). WIC was also shown to be protective against low birth weight depending on the women’s cervical health, particularly among women with incompetent cervices (OR: 0.2; 95% CI: 0.1–0.6). WIC was also protective against fetal death, especially among women with less than 12 years of education (OR: 0.2, 95% CI: 0.1–0.3).
	**Foster, E. M., Jiang, M., & Gibson‐Davis, C. M.** *The effect of the WIC program on the health of newborns*	2010	Mixed	USA	**Intervention:** Mother’s participation in the WIC program. **Study design:** Secondary analysis (using propensity scores to investigate the association between WIC participation and birth outcomes) of the Child Development Supplement (CDS) of Panel Study of Income Dynamics. **Study sample:** 3181 children and their mothers with data collected from 1997–2002	**Reported Outcomes**: Health outcomes. The study investigated birth outcomes, which includes birth weight, prematurity, maternal report of the infant’s health, small for gestational age, placement in the neonatal intensive care unit. Among the unmatched samples, children born to WIC-recipients had lower score on the maternal health rating of the child, were more likely to be born low birth weight, small for gestational age. Using a fixed effects model, preterm birth was significantly lower among WIC-participants (-0.07; p<0.05), birth weight (176.6g; p<0.01) and low birth weight (-0.09; p<0.01).
	Joyce, T., Racine, A., & Yunzal‐Butler*, C. *Reassessing the WIC effect*: *Evidence from the pregnancy nutrition surveillance system*	2008	Mixed	Florida, Georgia, Illinois, Indiana, Michigan, Missouri, New Jersey, North Carolina, Ohio, Virginia, USA	**Intervention:** WIC participation. **Study design:** Secondary analysis using data from 9 states that participated in the Pregnancy Nutrition Surveillance System from 1995–2004. **Study sample**: 2.8 million women who enrolled in WIC during pregnancy and recertified for WIC postpartum.	**Reported Outcomes:** Health outcomes. The study investigated birth outcomes, which include birth weight (in grams), low birth weight, very low birth weight, preterm birth, small for gestational age. Prenatal WIC participation is associated with decreases in rates of low birth weight (2.7 percentage points), very low birth weight (0.9 percentage points), and preterm birth (2.8 percentage points). Women who delay enrollment in WIC until the third trimester have rates of preterm birth that are 6.1 percentage points lower than postpartum enrollees and 4.8 percentage points less than first-trimester enrollees. However, given the presence of gestational age bias, the gains are more modest.
	**Khanani, I., Elam, J., Hearn, R., Jones, C., & Maseru, N.,** The impact of prenatal WIC participation on infant mortality and racial disparities	2010	Significant	Hamilton County, OH, USA	**Intervention:** WIC participation. **Study design:** Retrospective cohort study using data from WIC prenatal participants in Hamilton County, Ohio. **Study sample:** 18,091 women enrolled in WIC in the prenatal period.	**Reported Outcomes:** Health outcomes. White WIC participants were less likely to have preterm births compared to non-WIC participants (10.3% vs 8.7%, p = 0.004). African-American WIC participants were less likely to have preterm births compared to non-WIC participants (13.7% vs 20.0%, p<0.001).
	**Kim, K. & Frongillo, E.A.** *Participation in Food Assistance Programs Modifies the Relation of Food Insecurity with Weight and Depression in Elders*	2007	Significant	USA	**Intervention:** Participation in food assistance programs i.e. food stamps and home-delivered meals. **Study design:** Secondary analysis of the Health and Retirement Study (1996–2002) (HRS) and the Asset and Health Dynamics Among the Oldest Old dataset (1995–2002) (AHEAD).**Study sample:** 9481 from HRS and 6353 for AHEAD	**Reported Outcomes: Health outcomes** The study specifically investigated BMI and depression as outcomes. **BMI**: For AHEAD, food insecurity was positively associated to BMI among nonparticipants in the Food Stamp program, but not related to BMI among participants (p<0.004). Current food-insecure elders had higher BMI than current food-secure elders by 0.19 unit of BMI (p<0.033). **Depression**: Current food-insecure elders had higher depression scores than food-secure elders in both HRS (β = 0.27, p<0.001) and AHEAD β = 0.18, p<0.051).
	**Lazariu-Bauer, V., Stratton, H., Pruzek, R., & Woelfel, M. L.,** *A Comparative Analysis of Effects of Early Versus Late Prenatal WIC Participation on Birth Weight*: *NYS*, *1995*	2004	Significant	New York state, USA	**Intervention:** Participation in the WIC program. **Study design:** Secondary analysis of dataset from the New York state WIC program. **Study sample:** Mother-infant pairs comprising of infants born in New York State in 1995 whose mothers participated in the WIC program during their pregnancy. 77,601 records were available for analysis.	**Reported Outcomes**: Health outcomes. Infants born to WIC participants who enrolled early were 70g heavier on average than those who enrolled late. Black and Hispanic full-term infants experienced larger WIC effects than Whites (79, 75, 43 g, respectively). Effects of longer prenatal WIC participation were greatest for the inadequate prenatal care group (83 g).
	**Leung, C. W., Willett, W. C., & Ding, E. L.,** *Low-income Supplemental Nutrition Assistance Program participation is related to adiposity and metabolic risk factors*	2012	Non-significant	USA	**Intervention:** Participation in the Supplemental Nutrition Assistance Program (SNAP). **Study design:** Cross sectional analysis of NHANES data 2003–2006**. Study sample:** 2250 low income adults	**Reported Outcomes:** health outcomes. SNAP participation was positively associated with obesity (PR: 1.58), waist circumference in men (PR: 2.04), and waist circumference in women (PR: 2.95).*PR = prevalence ratio
	**Muhajarine, N., Ng, J., Bowen, A., Cushon, J., & Johnson, S.,** *Understanding the Impact of the Canada Prenatal Nutrition Program*: *A Quantitative Evaluation*.	2012	Significant	Canada	**Intervention:** Canada Prenatal Nutrition Program (CPNP)**,** a population-level health intervention that aims to contribute to improve health outcomes for pregnant women and newborn children under high-risk conditions. **Study design:** Observational cohort study. **Study sample:** 250,000 women who entered the CPNP program between 2002–2006.	**Reported Outcomes**: health outcomes. Participants with high CPNP exposure were less likely to have Pre-term birth .74(.65-.84), low birth rate .66(.60-.72), small for gestational age .89(.83-.96), and poor neonatal health .83(.78-.88) They were more likely to have babies large for gestational age 1.22 (1.11–1.35) all p <.05.
	**Nicholas, L. H.,** *Can Food Stamps help to reduce Medicare spending on diabetes*?	2011	Non-significant	USA	**Intervention**: Food Stamp receipt. **Study design**: Secondary analysis using longitudinal data from the Health and Retirement Study (HRS) linked to Medicare claims data. **Study sample**: 30,887 older Americans who were interviewed at least once between 1992–2006.	**Reported Outcomes:** Health outcomes and cost savings. There was no significant relationship between Food Stamp recipients and Medicare spending for older diabetics. (p>0.05)*There was no significant difference between hospitalization for diabetes or in outpatient utilization status between recipients and non-recipients (p>0.05).*Exact p-value not reported.
	**Rimmer, J. H., Wang, E., Pellegrini, C. A., Lullo, C., & Gerber, B. S**., *Telehealth weight management intervention for adults with physical disabilities*: *a randomized control trial*.	2013	Significant	Alabama, USA	**Intervention:** Low-cost telephone intervention supported with a web-based remote coaching tool called POWERS. POWERS contains the following sections: health appraisal, goal setting, implementation strategies, notes, and links to health promotion materials. Another arm called POWERSplus comprised of coaching and provision of nutritional information. **Study design:** Randomized controlled trial. **Study sample:** 102 people with physical disabilities (spinal cord injury, multiple sclerosis, spina bifida, cerebral palsy, stroke, or lupus).	**Reported Outcomes:** Health outcomes. There was a significant group and time interaction (P < 0.01) in post-intervention body weight. Both the POWERS and POWERSplus groups demonstrated greater reduction in body weight compared with the control group (POWERS: -2.1 ± 5.5 kg, -2.4 ± -5.9%; POWERSplus: -0.5 ± 5.0 kg, -0.6 ± 4.3%; control: +2.6 ± 5.3 kg, 3.1 ± 7.4%).
	**Ver Ploeg, M., Mancino, L., Lin, B. H., & Wang, C. Y.,** *The vanishing weight gap*: *trends in obesity among adult food stamp participants (US)(1976–2002)*.	2007	Non-significant	USA	**Intervention:** USDA’s Food Stamp Program. **Study design:** Secondary analysis from NHANES (1976–1980, 1988–1994, 1999–2002).**Study sample:** 20,845 observations	**Reported Outcomes**: Health outcomes. The association between food assistance program participation and body weight measures has decreased over the past 30 years. Specifically, among white women, the results showed that there was no significant difference in BMI or probability of overweight/obesity between food stamp participants and eligible nonparticipants.
**Income Support**	**Arno, P. S., Sohler, N., Viola, D., & Schechter, C.** *Bringing health and social policy together*: *The case of the earned income tax credit*	2009	Significant	USA	**Intervention**: Earned income tax credit (EITC) program **Study design**: Secondary analysis using data from the Annual Social and Economic Supplement to the 2001 Current Population Survey (CPS).**Study sample**: Children in households headed by an unmarried woman, or a married woman with absent spouse, with low/moderate incomes (<$30,000).	**Reported Outcomes**: Health outcomes. Each percentage point increase in EITC penetration (within or between states) is associated with a 23.2 per 100,000 *reduction* in infant mortality rate (P = 0.013). Among mothers who were not eligible for the credit, 75% reported all of their children lacked health insurance coverage. However, among mothers who were eligible for the EITC, 54% reported all of their children lacked health insurance coverage (P< 0.00005). Single mothers with low or moderate incomes who were ineligible for the EITC program were 1.4 times more likely to lack health insurance for all of their children than single mothers who were eligible to receive the credit.
	**Frank, D. A., Neault, N. B., Skalicky, A., Cook, J. T., Wilson, J. D., Levenson, S., … & Berkowitz, C**. *Heat or eat*: *The Low Income Home Energy Home Assistance Program and Nutritional and Health Risks among Children Less than 3 Years of Age*	2006	Significant	Baltimore, MD; Boston, MA; Little Rock, AK; Los Angeles, CA; Minneapolis, MN; Washington, DC	**Intervention:** Low Income Home Energy Assistance Program (LIHEAP). **Study design:** Cross-sectional. **Study sample:** 7074 caregivers with children < 3 years of age with household assistance and food insecurity from Jun 1998 –December 2004.	**Reported Outcomes: Health outcomes and cost savings.** (Height/weight as proxy for undernutrition, and recent emergency department admissions.) Children aged 2–3 years in recipient households were not more likely to be overweight (BMI ≥95th percentile) than those in non-recipient households (AOR: 0.83; 95% CI: 0.46–1.49). Rates of age-adjusted lifetime hospitalization—excluding birth and the day of the interview—did not differ between recipient groups. Among the 4445 of 7074 children evaluated in the 2 EDs, children from households not receiving the LIHEAP had greater odds of acute hospital admission than those in recipient households (OR: 1.32 (1.00–1.75; p <.05).
	**Herd, P., Schoeni, R. F., & House, J. S.** *Upstream solutions*: *does the in the elderly*? *supplemental security income program reduce disability*	2008	Significant	USA	**Intervention:** Supplemental Security Income (SSI) **Study design:** Secondary analysis using data from the 1990 and 2000 censuses, employing fixed effects models, to test whether within-state changes in maximum SSI benefits over time leads to changes in disability. **Study sample:** People aged 65 and older.	**Reported Outcomes:** Health outcomes. Higher benefits are linked to lower disability rates. A $100 increase per month in the benefit was linked to a mobility limitation decrease of 0.46%. An increase of $100 in the maximum monthly state SSI benefit leads to a 1.8 percentage point decline in the probability of having a mobility limitation among low-income individuals.
	**Hogan, S. R., Speiglman, R., & Norris, J. C.,** *The effects of eliminating Supplemental Security Income*, *drug addiction and alcoholism eligibility on the mental health of low-income substance abusers*.	2010	Significant	California, USA	**Intervention:** Supplemental Security Income (SSI) **Study design:** Pre-post intervention, 42-month follow-up **Study sample:** 412 people 21–59 years of age	**Reported Outcome:** Health outcome (mental health status) Among those who retained SSI, there is a significant difference in psychiatric composite score of the addiction severity index (mean score of 0.45 vs 0.29, p<0.05). There is a significant difference between those who retained SSI and those who received no public assistance in receiving any mental health treatment (46.1% vs 36.3%, p = 0.029).
**Care coordination and community outreach**	**Bhaumik, U., Norris, K., Charron, G., Walker, S. P., Sommer, S. J., Chan, E., … & Woods**, *A Cost Analysis for a Community-Based Case Management Intervention Program for Pediatric Asthma*	2013	Significant	Boston, MA, USA	**Intervention:** Boston Children’s Hospital Community Asthma Initiative (CAI) **Study design:** Retrospective cost-benefit analysis. **Study sample:** 102 patients enrolled in the CAI program in the calendar year 2006.	**Reported Outcomes**: Cost savings. The CAI was associated with an adjusted ROI of 1.33 during the first 3 years, after controlling for other factors other than the CAI intervention. After adding benefits due to reduced school days a missed work day, the social ROI increased to 1.85.
	**Counsell, S. R., Callahan, C. M., Clark, D. O., Tu, W., Buttar, A. B., Stump, T. E., & Ricketts, G. D.,** *Geriatric Care Management for Low-Income Seniors*	2008	Mixed	USA	**Intervention:** Home-based care management for 2 years (Jan 2002 to Aug 2004) by a nurse practitioner and social worker who collaborated with the primary care physician and a geriatrics interdisciplinary team. **Study design**: Randomized controlled trial. **Sample type**: 951 adults aged 65 or older with an annual income of less than 200% of the federal poverty level.	**Reported Outcomes**: Cost savings. The cumulative 2-year ED vsiti rate per 1000 was lower in the intervention group than the control (1445 vs 1748, p = 0.03). Among the high risk of hospitalization patients, the ED (848 vs 1314, p = 0.03) and hospital admissions (396 vs 705, p = 0.03) rates were lower among the intervention group compared to the control. However, hospital admissions rate did not differ significantly between the two groups (700 vs 740, p-0.66).
	**Karnick, P., Margellos-Anast, H., Seals, G., Whitman, S., Aljadeff, G., & Johnson, D.,** *The Pediatric Asthma Intervention*: *A Comprehensive Cost-Effective Approach to Asthma Management in a Disadvantaged Inner-City Community*	2007	Significant	Chicago, IL, USA	**Intervention:** A combination of asthma education, reinforced education, and case management and reinforced education. **Study design:** Randomized controlled trial. **Sample type:** 212 children with asthma, aged 1 to 16 years.	**Reported Outcomes**: Cost savings. The average decline in utilization of health resources across all 3 intervention groups was significant: 69% for hospital days, 64% for ED visits, and 58% for clinic visits. Cost savings were greatest among the participants in the case management and reinforced education arm ($4503/person) compared to asthma education ($4021/person) or reinforced education ($4140/person).
	**Kothari, C. L., Zielinski, R., James, A., Charoth, R. M., & Carmen Sweezy, L. D,** *Improved Birth Weight for Black Infants*: *Outcomes of a Healthy Start Program*.	2014	Mixed	Kalamazoo, MI, USA	**Intervention:** Participation in the Healthy Babies Healthy Start Program (HBHS): a case management approach to home visitation. **Study design:** Secondary analysis (matched-comparison post-test only) of Michigan state- and Kalamazoo County-level birth certificate records for 2008–2010. **Sample type:** 9336 women who were residents of Kalamazoo County when they gave birth during the years 2008–2010, of which 1742 self-reported as Black and 7174 self-reported as White.	**Reported Outcomes**: Health outcomes. Black HBHS participants delivered higher birth-weight infants compared to Black non-participants (p = 0.05). However, there was no significant difference in birth outcomes between White participants and non-participant (p = 0.7 for birth weight; p = 0.55 for gestation).
	**Olds, D. L., Kitzman, H., Knudtson, M. D., Anson, E., Smith, J. A., & Cole, R.** *Effect of home visiting by nurses on maternal and child mortality*: *Results of a 2-decade follow-up of a randomized clinical trial*.	2014	Significant	Memphis, TN, USA	**Intervention:** Infant/toddler nurse home visiting beginning during pregnancy and continuing through child age 2 years. **Study design**: Randomized controlled trial. **Sample type**: 1138 women who were primarily African American women at less than 29 weeks of gestation, no previous live births, and with at least 2 of the following socioeconomic characteristics: unmarried, have less than 12 years of education, and/or unemployed.	**Reported Outcomes**: Health outcomes. The study investigated all-cause mortality in mothers and preventable-cause mortality in children. The mean 21-year maternal all-cause mortality rate was significantly different (p = 0.007) between control and treatment groups: 3.7% in the combined control groups (transportation/transportation + developmental screening) compared to 0.4% in the intervention group (transportation plus prenatal/postpartum home visiting for infants and toddlers).
	**Sadowski, L. S., Kee, R. A., VanderWeele, T. J., & Buchanan, D.,** Effect *of a Housing and Case Management Program on Emergency Department Visits and Hospitalizations Among Chronically IllHomeless Adults*	2009	Significant	Chicago, IL, USA	**Intervention:** Housing offered as transitional housing after hospitalization discharge, followed by placement in long-term housing; case management offered at primary study sites, transitional housing, and stable housing sites. **Study design:** Randomized controlled trial **Sample type:** 407 social worker referred homeless adults with chronic medical illnesses from Sept 2003 to May 2006.	**Reported Outcomes:** Cost savings. After adjusting for baseline covariates, the intervention group had a relative reduction of 29% in hospitalizations (95% CI: 10–44%), 29% in hospital days (95% CI: 8–45%), and 24% in ED visits (95% CI: 3–40%).
	**Song, Z., Hill, C., Bennet, J., Vavasis, A., & Oriol, N. E.** *Mobile clinic in Massachusetts associated with cost savings from lowering blood pressure and emergency department use*.	2013	Significant	Boston, MA, USA	**Intervention:** The Family Van, a mobile clinic based in the Boston area. **Study design:** Secondary analysis of a database with patient records from 1992-2009.**Sample type:** 5900 patients’ data were analyzed from Jan 2010 –Jun 2012	**Reported Outcomes**: Health outcomes and cost savings. Patients who presented with high blood pressure in the first visited experienced mean reductions of 10.7mmHg and 6.2mmHg in systolic and diastolic blood pressure respectively. These changes are associated with overall cost savings of $1.58 million in healthcare costs. The Family Van yielded a return on investment of 1.3.
	**Thomas, K. S., & Mor, V.,** *The Relationship between Older Americans Act Title III State Expenditures and Prevalence of Low-Care Nursing Home Residents*	2013	Significant	USA	**Intervention:** Old Americans Act registered service expenditures for the year 2000-2009.**Study design:** Retrospective cohort design, 10 years follow-up. **Study sample**: 14,485 low-care residents in nursing homes.	**Reported Outcomes**: Health outcomes and cost savings. Increased spending on home-delivered meals was associated with fewer residents in nursing homes with low-care needs. A decrease of 1% in number of low-care NH residents was associated with an additional $25 per person aged 65+ in the state.
	**Woods, E. R., Bhaumik, U., Sommer, S. J., Ziniel, S. I., Kessler, A. J., Chan, E., … & Nethersole, S.,** Community *asthma initiative*: *evaluation of a quality improvement program for comprehensive asthma care*.	2012	Significant	Boston, MA, USA	**Intervention**: Nurse case management and home visits with primary care and referral services, and nurse or nurse-supervised CHW home visits for asthma education, environmental assessment and remediation materials, and referral to IPM exterminator. **Study design:** Prospective cohort study. **Sample type:** 283 children aged 2–18 living in 4 urban zip codes, with asthma, who had hospitalizations or ED visits from Oct 1 2005 –June 30 2008	**Reported Outcomes:** Cost savings. At the 12-month mark of the study, there was a significant decrease in asthma ED visits (68.0%), any days of limitation of physical limitation (42.6%), patient (child) missed school (41.0%), and parent missed work (49.7%). There was a significant reduction in hospital costs compared with the comparison community (p<0.0001), with a return of investment of 1.46
**Other**	**Campbell, F., Conti, G., Heckman, J. J., Moon, S. H., Pinto, R., Pungello, E., & Pan, Y**. *Early childhood investments substantially boost adult health*	2014	Significant	Chapel Hill, NC, USA.	**Intervention**: Carolina Abecedarian Project, an early childhood development program aimed at promoting cognitive development among disadvantaged children (ABC). **Study design**: Cohort study with long-follow-up evaluated by randomization. **Study sample**: 111 children born between 1972 and 1977 who were living or in Chapel Hill, North Carolina	**Reported Outcomes**: Health outcomes (risk factors for cardiovascular disease, metabolic disease). Disadvantaged children randomly assigned to treatment have significantly lower risk factors for cardiovascular and metabolic diseases in their mid-30s, particularly among males. On average, systolic blood pressures between control and treatment group were significantly different (143mmHg vs 126mmHg, p = 0.018). One in four males had metabolic syndrome in the control group, compared to none in the treatment group (p = 0.007).
	**Fagg, J., Chadwick, P., Cole, T. J., Cummins, S., Goldstein, H., Lewis, H., … & Law, C.**, *From trial to population*: *a study of a family based community intervention for childhood overweight implemented at scale*	2014	Significant	United Kingdom	**Intervention**: Family-based weight intervention called MEND 7–13 (Mind, Exercise, Nutrition, Do it!)**Study design**: Pre/post intervention evaluation. **Study sample**: 9563 children who are overweight; and their family members	**Reported Outcomes**: Health outcomes (BMI and psychological distress). After adjustment, in the intervention group, BMI reduced by 0.76kg/m^2^ on average (p<0.0001), self-esteem score increased by 3.53U (p<0.0001), and psychological distress decreased by 2.64U (p<0.0001). These outcomes were less pronounced among children from less advantaged backgrounds and among Asians compared to white children.
	**Sacher, P. M., Kolotourou, M., Chadwick, P. M., Cole, T. J., Lawson, M. S., Lucas, A., & Singhal, A.** *Randomized controlled trial of the MEND program*: *a family‐based community intervention for childhood obesity*	2010	Significant	United Kingdom	**Intervention**: Mind, Exercise, Do It (MEND) **Study design**: Randomized controlled trial. **Sample type**: 116 Obese children (BMI ≥ 98^th^ percentile)	**Reported Outcomes**: Health outcomes. Participants in the intervention had a reduced waist circumference (-0.37; p<0.0001) and BMI (-0.24; p<0.0001) at 6 months when compared to controls. The program had an 86% attendance rate, suggesting that the program could be feasibly implemented in the community.

^1^ Results with a p-value less than 0.05 were considered significant.

### Housing Support

Overall, of 12 studies evaluating housing intervention, 4 studies– 3 from the US [20–22] and 1 from the UK [23]–reported both improved health outcomes and reduced health care costs. Five additional studies showed improvement in health outcomes including obesity and diabetes among women with children [24], asthma among adults [25], self-reported health status among adults [26], mobility among low income older adults [27] and HIV outcomes [28]. One study found that the provision of housing was significantly associated with lower health care spending among the chronically homeless with severe alcohol addiction [29]. One study with non-significant results pertained to health care costs as a result of a housing intervention that included the provision of housing and case management for homeless adults with chronic illness [30], and one study reported significantly negative results pertaining to health outcomes, which reported poorer mental health outcomes among the adolescent boys in the intervention group who were offered supportive housing in a neighborhood other than their own [18].

### Nutritional Support

Of the 11 studies related to nutritional support interventions, seven studies reported significantly improved health outcomes. Six studies were based in the US [31–36] and one was conducted in Canada [37]. No studies reported decreased health care costs associated with nutritional support interventions. Two studies reported null findings: one showed no significant relationship between food stamp recipients and the prevalence of diabetes, Medicare spending, or hospital utilization rates [38], and one reported no significant association between food assistance via a federally-funded nutritional assistance program and probability of overweight/obesity [39]. One study[19] reported significantly increased obesity associated with food assistance in a sample of adult SNAP recipients, and one study [40] reported mixed results, showing that participation in the Special Supplemental Nutrition Program for Women, Infants and Children (WIC) was associated with significantly higher birth weights, but this result was sensitive to model estimation parameters such that this result was only observed in fixed effects models.

### Income Support

We found four studies related to income support, all of which demonstrated a positive relationship between income support interventions and health outcomes, or both health outcomes and health care costs. A total of three studies reported Supplemental Security Income had a positive effect on health outcomes including infant mortality [41], disability rates among the elderly [42], and mental health among former beneficiaries [43]. We found one study[44] that showed both improved health outcomes and decreased health care costs; the Low Income Home Energy Assistance Program (LIHEAP) was associated with decreased probability of overweight and obesity among children and lower hospital admission rates.

### Care coordination and community outreach

Of the nine studies we reviewed with care coordination and community outreach interventions, four showed decreased health care costs associated with the intervention [45–48]. Two other care coordination interventions were shown to have a significantly positive impact on health outcomes, specifically on all-cause mortality in mothers [49] and on birth-weight among African American mothers [50], and two community outreach studies were associated with lower health care costs and better health outcomes [51, 52]. In these cases, community outreach interventions included a mobile health clinic and home-delivered meals. We also found one study with mixed results, showing that care coordination interventions reduced health care costs, but did not significantly impact quality-of-life related health outcomes [53].

### Other

We found three studies related to interventions with major educational components that were associated with improved health outcomes, especially among children. Two of these studies were based on the MEND trial, a multicomponent trial comprised of educational and physical activities designed to combat obesity among children aged 7–13 years [54, 55]. The third study was related to the Carolina Abecedarian Project, a program that was originally designed to promote cognitive development among disadvantaged children, which was ultimately shown to lower risk factors for cardiovascular and metabolic diseases among participants [56].

## Discussion

Our analysis of the literature indicates that several interventions in the areas of housing, income support, nutritional support, and care coordination and community outreach have had positive impact. These interventions should be of interest to health care policymakers and practitioners seeking to leverage social services to improve health or reduce costs. Importantly, 100% of the studies evaluating income support programs, 88% of the care coordination and community outreach interventions, 83% of the housing support programs, and 64% of the nutritional support programs evaluated had statistically significant, positive effects on health outcomes alone or on both health outcomes and health care spending. Furthermore, the direction and magnitude of the results were robust across different study designs. Among randomized control trials and quasi-experimental studies, 85% of interventions showed positive health impacts or reductions in health spending. Overall, a small number of studies met criteria to be included in our sample. This not only suggests the need for additional interventional research addressing both health and cost outcomes, but also points to the opportunity for pushing the existing body of evidence on population health to the next level of intervention development.

Findings from this work, the majority of which was conducted with low-income populations, suggest that keeping a population healthy may require medical providers to link with unconventional partners such as housing authorities, food banks, and schools. The inclusion of researchers in cross-sector partnerships to document impact with empirically sound methods may further sustain and ultimately help to scale interventions targeted at the social determinants of health. Moreover, while case managers and care coordinators have become a ubiquitous feature of many health care systems, the literature provides an impetus for potentially expanding the scope of services that case managers and care coordinators manage. Careful consideration of these issues may be particularly prudent among health systems that have transitioned to value-based financing or accountable care models, where health outcomes have been explicitly prioritized in performance metrics.

The literature highlights the “wrong pocket problem” [57], in which the savings that accompany health improvements do not accrue to the investor. In economic circles, this challenge is more commonly termed an externality. Many social service interventions (e.g., income support, housing) generate positive health outcomes, yet social service sectors receive little if any reward for their contribution to the creation of health in the population. Similarly, depending on its payer and contract mix, a health care organization that contributes to a person’s health does not reap the full social benefit from those health improvements. Thus, the wrong pocket problem discourages cross-sector collaboration when in fact the literature reviewed here suggests a high degree of mutual dependence and potential reward from coordinated health care and social services. These are questions we can and should be wrestling with more explicitly, particularly as literature like this empirically demonstrates the broad range of inputs required to create health.

Despite the substantial consistency of the findings overall, several limitations and gaps were apparent in the literature. First, the number of studies that examine impact on health care spending was relatively modest. As policymakers, payers, and providers seek to support programs that address the social determinants of health, understanding the health care cost offsets will be critical for widespread endorsement. Second, few studies examine interventions related to transportation services, public safety, education, and income support programs; and the majority of studies examine impacts on low-income groups, limiting the generalizability of findings. Third, several studies would be strengthened by better comparison groups, larger samples, and more sophisticated analytical methods to address potential confounding influences. These limitations of the literature, continue to hamper the translation of these findings into policy or practice [58].

In summary, we found substantial evidence of improved health outcomes and/or reduced health care spending related to interventions that addressed housing, nutrition, income support, and care coordination and community outreach needs. At the same time, this literature can be improved in scope and rigor. Further studies, particularly examining a broader set of interventions with methods to determine causal effects on both health outcomes and health care spending, are needed to produce a comprehensive understanding of the degree to which interventions to address the social determinants of health care improve health and reduce health care costs.
